# DNA G-quadruplex structures: more than simple roadblocks to transcription?

**DOI:** 10.1093/nar/gkab609

**Published:** 2021-07-13

**Authors:** Jenna Robinson, Federica Raguseo, Sabrina Pia Nuccio, Denise Liano, Marco Di Antonio

**Affiliations:** Imperial College London, Chemistry Department, Molecular Sciences Research Hub, 82 Wood Lane, London W12 0BZ, UK; Institute of Chemical Biology, Molecular Sciences Research Hub, 82 Wood Lane, London W12 0BZ, UK; Imperial College London, Chemistry Department, Molecular Sciences Research Hub, 82 Wood Lane, London W12 0BZ, UK; Institute of Chemical Biology, Molecular Sciences Research Hub, 82 Wood Lane, London W12 0BZ, UK; Imperial College London, Chemistry Department, Molecular Sciences Research Hub, 82 Wood Lane, London W12 0BZ, UK; Institute of Chemical Biology, Molecular Sciences Research Hub, 82 Wood Lane, London W12 0BZ, UK; Imperial College London, Chemistry Department, Molecular Sciences Research Hub, 82 Wood Lane, London W12 0BZ, UK; Institute of Chemical Biology, Molecular Sciences Research Hub, 82 Wood Lane, London W12 0BZ, UK; Imperial College London, Chemistry Department, Molecular Sciences Research Hub, 82 Wood Lane, London W12 0BZ, UK; Institute of Chemical Biology, Molecular Sciences Research Hub, 82 Wood Lane, London W12 0BZ, UK; The Francis Crick Institute, 1 Midland Road, London NW1 1AT, UK

## Abstract

It has been >20 years since the formation of G-quadruplex (G4) secondary structures in gene promoters was first linked to the regulation of gene expression. Since then, the development of small molecules to selectively target G4s and their cellular application have contributed to an improved understanding of how G4s regulate transcription. One model that arose from this work placed these non-canonical DNA structures as repressors of transcription by preventing polymerase processivity. Although a considerable number of studies have recently provided sufficient evidence to reconsider this simplistic model, there is still a misrepresentation of G4s as transcriptional roadblocks. In this review, we will challenge this model depicting G4s as simple ‘off switches’ for gene expression by articulating how their formation has the potential to alter gene expression at many different levels, acting as a key regulatory element perturbing the nature of epigenetic marks and chromatin architecture.

## Graphical Abstract

**Graphical Abstract:**
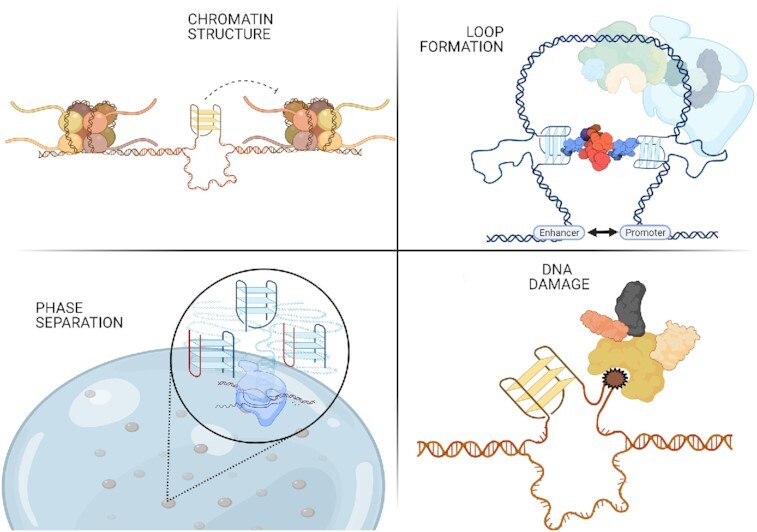
DNA G-quadruplexes act as transcriptional hubs by means of diverse mechanisms, including: modulation of chromatin structure, regulatory protein recruitment and formation of DNA loops, stimulation of liquid-liquid phase separation and eliciting DNA damage and repair.

## INTRODUCTION

**Figure 1.**
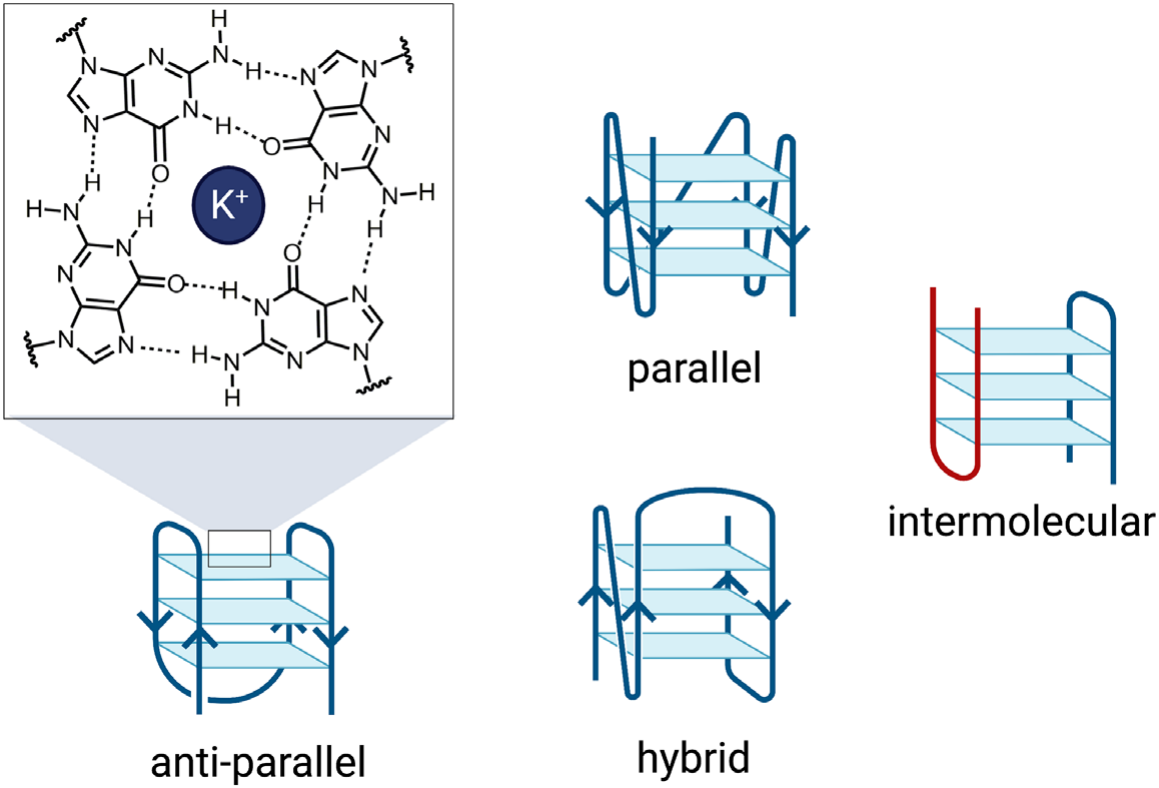
Schematic representation of G-quadruplex structures. G4s are constituted of four guanine bases arranged in a square planar conformation (G-tetrad) held together by Hoogsteen hydrogen bonding and further stabilized by alkali cation such as K^+^. Specific G4 topologies can be formed and include anti-parallel, parallel and hybrid structures, depending on the relative orientation of the DNA strand within the structure. Intermolecular G4 structures can also be formed when more than one DNA strand is used to generate the final structure (red and blue strands).

G-quadruplexes (G4s) are DNA structures that can arise under physiological conditions from guanine-rich DNA sequences (Figure 1) (1–4). Although the ability of guanine analogues to assemble into tetrameric structures has been known since 1962 (5), the idea that G4 structures could form in the context of genomic DNA was only seriously considered 40 years later when the crystal structure of a G4 formed by the human telomeric DNA sequence was reported for the first time (6). The very same year a different G4-forming sequence was described in the promoter region of the oncogene *MYC* by Hurley *et al.* (7). Importantly, this study was not limited to the structural characterization of the quadruplex, but also provided evidence that such a G4 was biologically active by showing MYC-downregulation as a direct consequence of G4 stabilization by the porphyrin-based ligand TMPyP4 (7). This observation sparked interest in non-telomeric G4s and suggested that these structures could act as transcriptional repressors, a concept supported in several following studies on G4s formed in other oncogene promoters, such as c-KIT, BCL-2, KRAS and VEGF (8). This general notion was further consolidated by multiple bioinformatic studies revealing that G4s are enriched at gene promoters, particularly in oncogenes (9–11), supporting a role of these structures in regulating gene-expression and their potential to be targeted by small-molecules for therapeutic intervention.

However, most of the initial studies that portrayed G4s as antagonists to gene expression relied on the use of synthetic molecules as G4-stabilizers or *in vitro* models of transcription (12), typically plasmid-based, which do not take into account the complexity of endogenous gene expression within chromatin. Indeed, direct evidence to support the idea that endogenous G4 formation at gene-promoters results in transcriptional repression is still lacking. In 2012 Rodriguez *et al.* challenged the notion of G4s as direct transcriptional repressors, by revealing that treatment with the potent G4 ligand pyridostatin (PDS) caused DNA-damage at G4 sites in a transcription and replication dependent fashion (13). In this key study, which generated the first genome-wide G4 map, it was demonstrated that the DNA damage elicited by PDS at G4 sites caused gene down-regulation, rather than the formation of the G4 structure itself. The same year, Hurley and co-workers challenged their own observations made with TMPyP4 10 years earlier, reporting how treatment with G4 ligands could lead to MYC downregulation without direct targeting and stabilization of the MYC G4, but rather as an indirect consequence of global G4 stabilization elicited by the ligand (14).

More recently, the development of G4-selective antibodies has enabled genome-wide mapping of G4s by immuno-precipitation experiments followed by sequencing (ChIP-Seq). These studies have allowed for a far more direct investigation of the association between endogenous G4-formation and gene expression, revealing an almost inverted picture to the earlier investigations. Indeed, G4 ChIP-Seq performed in keratinocytes (15) and human xenografts (16) using the G4-selective antibody BG4, has demonstrated that G4s are prevalently found at gene promoters of transcriptionally active genes, acting as transcriptional enhancers rather than repressors. The link between G4 formation and active transcription has been confirmed beyond keratinocytes, in liposarcoma cells still using BG4-based ChIP-Seq (17), as well as in a range of mammalian cell lines using a G4-selective peptide probe (18), and even with single-molecule detection of G4s within live cells under non-perturbative conditions (19). Furthermore, G4 formation seems to contribute to activated gene expression by means of many different mechanisms that span from transcription factor binding (20) to guanine oxidation (21). Despite this, there is still a misrepresentation in the scientific community of G4s acting as transcriptional roadblocks and repressors, which is reminiscent of the initial hypothesis postulated 20 years ago. In this review, we aim to provide a critical assessment of the latest research relating G4s to transcriptional regulation, discussing the genome-wide effects of G4 formation on chromatin architecture, long-range interactions, phase separation and DNA oxidation.

### G4 formation in the chromatin context

**Figure 2.**
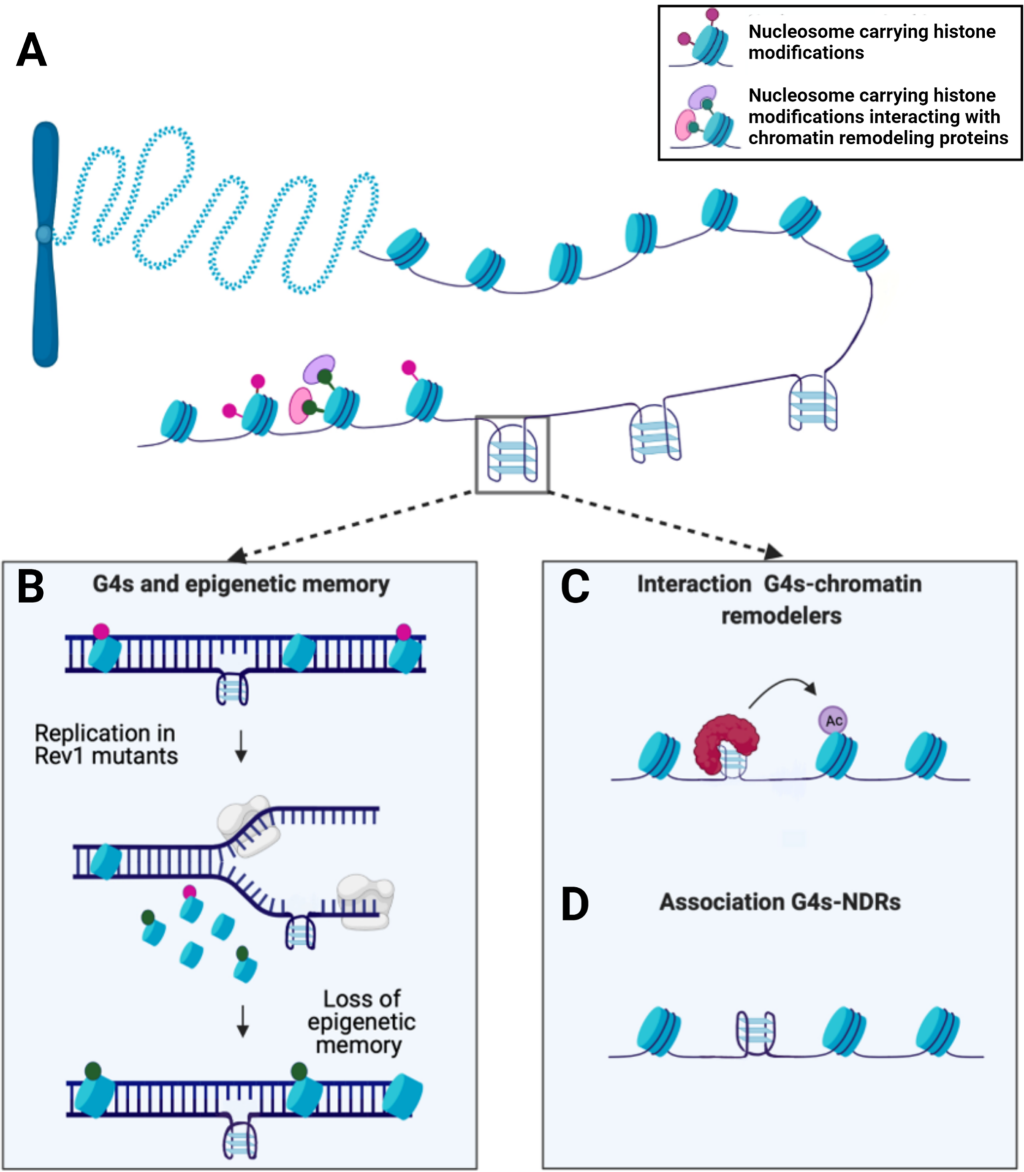
G4s and chromatin structure. (**A**) Structure of chromatin comprised of DNA wrapped around nucleosome complexes. (**B**) REV1 mutants unable to resolve G4s during DNA replication exhibit altered histones modifications in the newly synthesized strand and a consequent loss of epigenetic memory. (**C**) G4s can interact with a wide panel of proteins, including chromatin remodelers such as BRD3. (**D**) G4s are strongly associated with nucleosome depleted regions (NDRs) as confirmed by BG4-ChIP.

To fully appreciate and investigate the role G4 formation may play in gene regulation, we need to consider the genomic context in which G4s exist. The 3D organization of genetic material in cells presents a high level of complexity, where the canonical double helix of DNA is wrapped around proteins in a macromolecular structure known as chromatin. Chromatin is made of nucleosomes: a repetitive unit consisting of histone proteins which wrap around a stretch of DNA (ca. 146 bp) contributing to the folding of DNA inside the nucleus (Figure 2A) (22,23). However, genome sequencing and microarray hybridization technologies have revealed that nucleosome occupancy is not homogenous across DNA and that there are certain genomic regions depleted of nucleosomes (24). Nucleosome depleted regions (NDRs) are stretches of more accessible DNA which act as hubs for protein binding, including those that regulate transcription (24). Interestingly, the location of NDRs within the genome is highly dynamic and dependent on chemical modifications to histones, such as methylation or acetylation, which change the interaction of histones with key proteins involved in regulating gene expression (25). Therefore, nucleosome positioning across the genome is key to define the epigenetic status of a cell and this organization appears to also be associated with the formation of certain DNA secondary structures, including G4s.

The first evidence that G4s may have a role in shaping chromatin was highlighted in a study focused on REV1, a protein belonging to the Y family of DNA polymerases, that has an essential role in ensuring replication proceeds when DNA damage occurs (26). Cells containing a mutant form of *REV1* were characterized by delayed or fully compromised DNA replication, particularly at G4-forming sequences (26). The unresolved G4 structures in *REV1* mutants led to replication fork stalling, but surprisingly also had a significant impact on the epigenetic status of the cell (Figure 2B). In particular, an increase in the expression of the p-globin locus was observed. This upregulation was due to the loss of a histone modification negatively associated with transcription: H3K9 dimethylation, as well as incorporation of newly acetylated histones around the G4-forming site, promoting active transcription.

However, a later study of a separate locus revealed that unresolved G4 structures in *REV1* mutants could also lead to the *loss* of distinct histone marks that promote transcription, suggesting that the role of G4s in modifying the histone code is dependent on their genomic context (27). Although, these findings were limited to chicken DT40 cells, they suggested for the first time a relationship between G4s and the installation of epigenetic marks, which was more recently confirmed by immunofluorescence studies (28). In this latest work, colocalization between G4 structures and histone modifications found in nucleosome-depleted euchromatin regions was observed, underlining the potential of G4s in moulding the epigenetic landscape.

From these findings, some open questions arise: are G4s a passive element in the deposition of histones marks or do they have a mechanistic role in shaping chromatin that is not limited to replication stalling? To unravel any mechanistic link between G4s and chromatin structure, it may be key to investigate G4-interacting proteins especially those that are known for having an active role in chromatin remodelling. An example is the protein BRD3 that was found to be one of the top hits in an unbiased microarray screening of the G4-interactome (29). BRD3 contains a bromo-domain capable of binding acetylated histones, allowing for the recruitment of RNA polymerase and initiation of transcription (30). The idea that G4s interact with BRD3 within cells has been substantiated by BG4 ChIP-seq and BRD3 ChIP-seq analysis, which revealed a significant colocalization of G4 motifs within BRD3 occupancy sites (31). Additionally, the interaction between G4s and BRD3 does not prevent its binding to acetylated histones or other chromatin remodelers, suggesting that BRD3-G4 interactions might instead guide the recruitment of chromatin remodelling complexes to favour transcription at G4-sites (Figure 2C).

These findings highlighted the relevance that G4s may have in engaging key regulators of chromatin architecture, leading to the hypothesis that global changes in chromatin structure could be directly caused by G4 formation in the genome. This hypothesis is supported by bioinformatic associations made on a genome-wide scale, which demonstrated that G4-forming sequences are highly enriched at nucleosome depleted regions (Figure 2D) (10,32). Although these first studies were based on computational predictions of G4s, such results have recently been validated directly in the chromatin context by using the G4-selective antibody BG4 which confirmed the enrichment of G4s at nucleosome free sites (15). Specifically, 98% of G4s identified by BG4-ChIP coincided with NDRs, an observation that has now been recapitulated in independent studies (17). This exemplifies the interconnectedness between G4 formation and chromatin structure and strongly suggests that these structures might mark transcriptionally active regions and possibly influence nucleosome positioning.

Despite the fact that there is now significant evidence suggesting that G4s may act as an active factor in shaping the structure of chromatin, for example by altering histone modifications and nucleosome placement, it is also conceivable that G4s form at open chromatin sites as a simple consequence of DNA accessibility and negative supercoiling that is also required for active transcription (33). Future experiments must focus on assessing a direct causation between chromatin remodelling and G4 formation, underpinning whether these structures can act as recruiters of epigenetic enzymes to actively shape chromatin structure. Previous reports on BG4 ChiP-Seq have suggested that accessible chromatin is necessary but not sufficient for G4s to form (15), which indicates that these structures do not form as a simple consequence of chromatin accessibility. Identifying the regulators of their formation within open chromatin sites will be key to underpin the relevance of G4s in moulding the epigenetic landscape.

### G4 formation stabilizes R-loops

**Figure 3.**
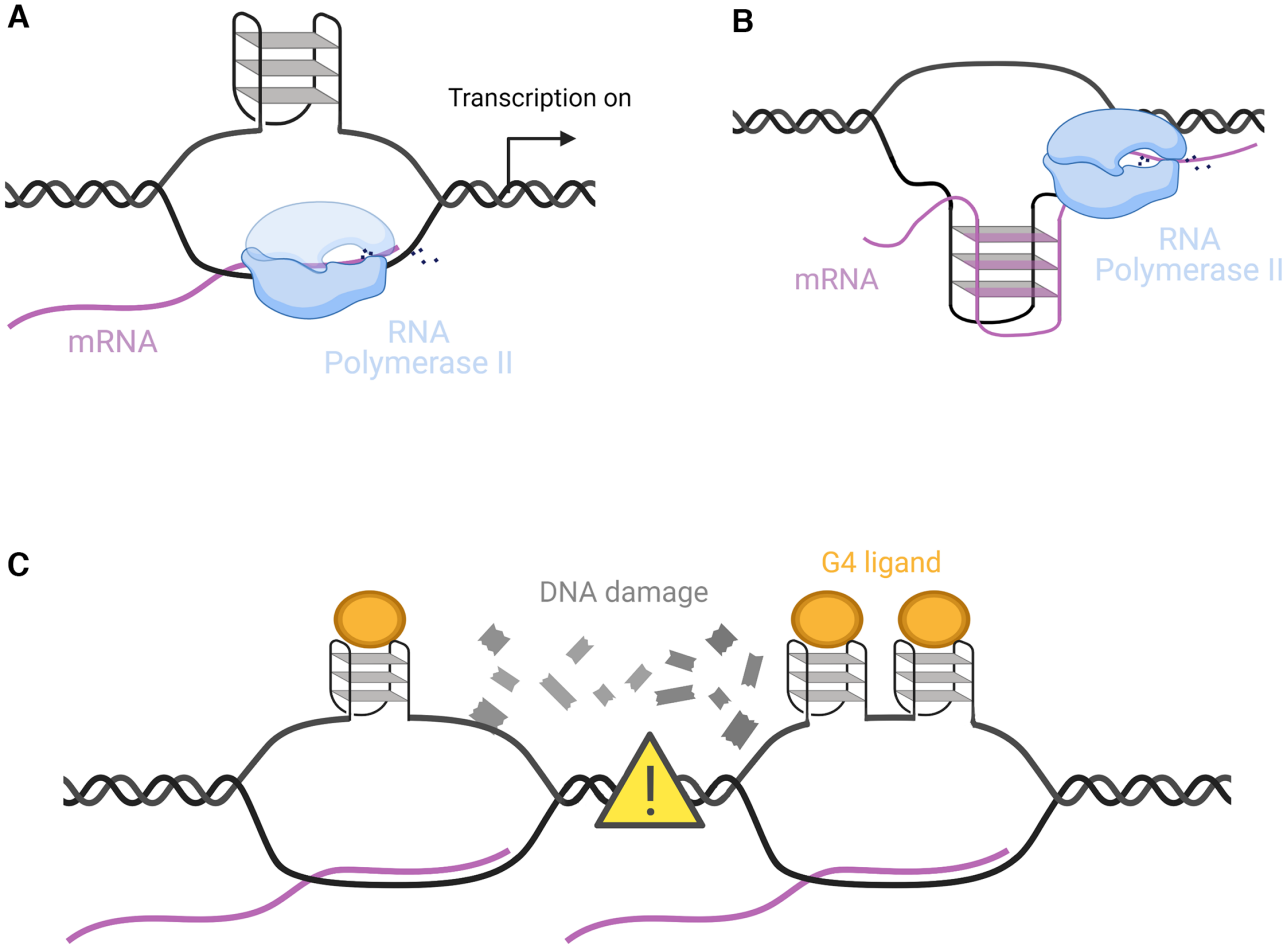
G4s and R-loops. (**A**) Intramolecular G4s formed on the non-template strand stabilize R-loops and increase transcriptional output *in vitro*. (**B**) Intermolecular DNA:RNA hybrid G4s may also form stabilizing R-loops on the template strand. (**C**) The use of G4-stabilizing small-molecules increases the prevalence of G4s and R-loops within cells. This can lead to the collision of nearby unscheduled R-loops, resulting in DNA-damage which hinders transcription.

In addition to nucleosome free DNA, a further structural requirement of transcription is the ability of duplex DNA to be unwound into single strands, one of which acts as the template strand for RNA polymerase, whilst the other is denoted the non-template strand. As polymerase transcribes the template strand, a three-stranded intermediate is formed comprising the two original DNA strands and the newly transcribed mRNA sequence, which make up a so-called R-loop (Figure 3A) (34). Interestingly, the requirements for R-loop generation are also compatible with G4-formation, as RNA:DNA hybrids form most stably with C-rich regions on the template strand of DNA (35). Thus, the creation of R-loops often displaces a single-stranded G-rich sequence which may be primed for folding into a G-quadruplex (36–39). This can occur from the formation of an intramolecular G4 on the displaced strand (Figure 3A) or a DNA:RNA hybrid intermolecular G4 (Figure 3B) (40,41).

Such R-loops form significantly in actively transcribed genes and interestingly, genome-wide studies have also confirmed formation of G4-structures in transcriptionally active genes (15,17,18). Furthermore, it appears that G-quadruplexes may have a functional role in mediating transcription by actively stabilizing the R-loop (42), allowing for enhanced transcription. A recent study demonstrated this by showing that the placement of G4s on the non-template strand enhances R-loop formation resulting in significantly increased transcript output, RNA polymerase initiation and elongation (43). This study also revealed that such an effect was strand dependent, as no such increases in transcription are seen when the G4 motif was found on the template strand. Despite these interesting results, this work was limited to *in vitro* measures of transcription which may not encapsulate the complex interactions of G4s within cells. Thus, it would be worth further investigating if the presence of G4s in R-loops is similarly correlated with increased transcriptional output within a chromatin DNA context that is more representative of transcriptional processes in living cells.

In contrast to this work suggesting G4s have a positive effect on transcription by stabilizing R-loop formation, it has been noted that over-stabilization of G4s can have deleterious effects on transcription. For instance, artificially enriching G4 content either by exposing cells to G4-ligands (44) or down-regulating G4 helicases (42), increases the number of R-loops due to the positive relationship between G4 and R-loop formation (Figure 3C). These unscheduled R-loops can collide and stimulate DNA-damage in the form of double strand breaks as demonstrated by 53BP1 and γH2AX marks that are enriched after G4 ligand incubation. In turn, such DNA damage and transcription-replication fork collisions can significantly hinder successful transcription, particularly in cells deficient in DNA damage repair proteins such as BRCA2 which are also known to be highly sensitive to G4 ligands (44,45). This is a useful example of how disrupting the natural homeostasis of G4 formation within cells can have antagonistic effects on gene expression that are not otherwise observed, highlighting the need to distinguish between such studies and those considering the epigenetic effects of endogenously formed G4s.

### G4 formation can promote long-range DNA interactions

Although the enrichment of G4s in promoters of highly transcribed genes sprouted the notion that G4s may mediate transcriptional control (potentially by shaping local chromatin structure and R-loop formation), it is possible that this role expands beyond the local context and into additional long-range mechanisms of epigenetics. In fact, considering promoter sites directly proceeding genes constitute only a fraction of the human genome, research in this area may represent just the tip of the iceberg when it comes to investigating how G4s control gene expression. For example, a single gene may have its transcription regulated by multiple regulatory sites littered all throughout the genome—sites which are able to exert their regulatory powers over long-distances and are often dysregulated in disease states (46–48). In recent years, compelling research has been conducted suggesting that G4s may not only be involved in proximal transcriptional control, but part of these long-range mechanisms that define an important component of the cell's epigenetic toolkit.

**Figure 4.**
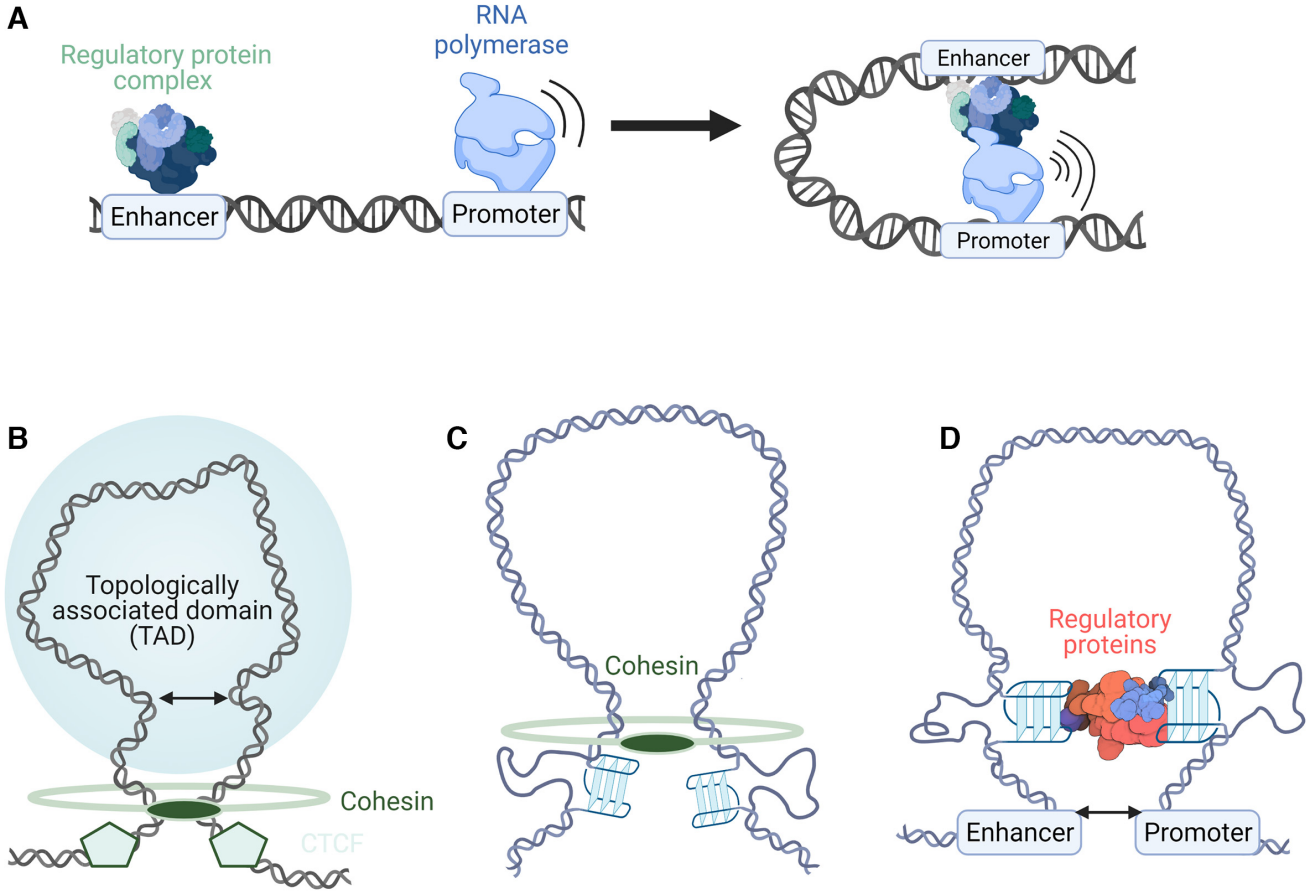
G4s in enhancer-promoter loop formation. (**A**) Schematic of how enhancers interact with their respective promoters to increase polymerase activity and transcription. (**B**) Cohesin and CTCF cooperate to form stable loops (topologically associated domains) in DNA. (**C**) G4 enrichment at loop boundaries possibly stalls cohesin. (**D**) G4s act as recruiters of regulatory proteins such as transcription factors that stabilize loops.

One of the most important long-range interactions utilized for epigenetic control is between transcriptional promoter and enhancer sites of DNA (Figure 4A) (49). While promoters mark the beginning points of transcription—the region at which RNA polymerase binds and begins its journey transcribing the code of DNA—enhancers act as auxiliary regulatory regions, recruiting additional proteins to carefully control the extent to which genes are expressed (50). This enhancer-mediated control occurs through the binding of large protein complexes containing transcription factors and cofactors, which increase the ability of RNA polymerase II to initiate and sustain transcription (50). So although promoter regions are sufficient to achieve a basal level of gene expression (51), it is through the utilization of enhancers that increases of transcription as high as 100-fold can be achieved (52). Interestingly, this control of transcription can occur over hundreds of thousands of base pairs; (53) thus it has long been speculated that distal interactions in DNA may be facilitated by the folding of DNA into secondary structures such as loops to allow normally separated sequences to meet (Figure 4A) (49).

To test this hypothesis, countless chromatin conformation capture (3C/Hi-C) experiments have been conducted, which allow for the cross-linking and downstream sequencing of DNA regions known as ‘topologically associated domains’ (TADs) that are connected not in sequence, but in space (54). The results of which have shown that DNA looping occurs across the genome and can allow for distal interactions between regulatory sites. However, this work also revealed that regions where loops form in the genome are in fact not random, but instead occur in a sequence-specific fashion (55). The mechanism for this specificity is widely agreed to be achieved through the cooperation of two proteins: cohesin and CTCF (Figure 4B). Cohesin is a ring-like protein, initially discovered for its role clasping together sister chromatids during replication; however, it has a secondary function translocating down stretches of DNA to form transient loops (56). In order to prevent cohesin sliding down DNA indefinitely, the cell has utilized cohesin stop points in the form of binding to the transcription factor CTCF which is named after its interaction with CCCTC sequences in DNA. CTCF binds to and blocks the releasing function of cohesin (57), which means its presence defines the start and end points of loops and in turn where the boundaries lie for topologically associated domains.

However, CTCF binding motifs are not the only sequences enriched at loop boundaries as G-quadruplex sequences are also abundant at the edges of loops (58). Moreover, it was found that the presence of G4s at loop boundaries increases the stability of DNA loops and in turn enhances long-distance DNA interactions (58). Although the mechanism for this correlation has yet to be fully explored, the authors of this study speculated that G4s may act analogously to CTCF, stalling the progression of cohesin and thus defining the boundaries of distal interactions (Figure 4C). This is further supported by the fact that G4 motifs at loop boundaries are significantly enriched on the same strand as the CTCF binding motif, suggesting some cooperation between the action of CTCF and G4 formation (58). This work demonstrates, perhaps counter-intuitively, that the protein-stalling capabilities of G4s that have been demonstrated *in vitro* with RNA polymerase, may *in vivo* be used to enhance rather than inhibit transcription and polymerase activity by enriching loop formation. Furthermore, these findings highlight even more how the simple strategy of considering individual G4 formation at specific gene promoters might significantly underestimate the potential roles of G4s in the regulation of gene expression.

Beyond the initial extrusion of enhancer-promoter loops in DNA, G4s may further stabilize these loops by promoting the binding and recruitment of key regulatory proteins which inhibit loop collapse. It is possible that this is done somewhat indirectly as G4s are correlated with more open, accessible chromatin and also reduced DNA methylation both of which promote protein-DNA interactions (59,60). However, various *in vitro* studies have demonstrated the high affinity of multiple regulatory proteins for G4 structures including, eukaryotic transcription factors SP1 (61), MAZ (62) and YY1 (63) and viral regulatory proteins such as the transcription factor ICP4 (64). Additionally, proteins that form part of large transcription-enhancing complexes such as the transcriptional co-activator BRD3 (29,31) and the chromatin regulating protein PARP-1 (65) have been shown to interact strongly with G4s. Within cells, enhancers and promoters containing G4-forming sequences additionally have significantly higher levels of transcription factor binding, which is accompanied by increased levels of RNA pol II occupancy and transcriptional activity (17,20,58). This enrichment is seen even when controlling for differences in chromatin accessibility (58) and the G-richness (20) of a given DNA region suggesting that it is the formation of the G-quadruplex structure itself that is responsible for enhanced transcription, as opposed to a simple correlation with open chromatin or GC content.

These findings support a model of G-quadruplex structures as scaffolds for protein binding, which may promote both local and remote interactions of gene regulation sites (Figure 4D). This hypothesis has been substantiated further with work studying the interactions of G-quadruplexes with the transcription factor ying-yang 1 (YY1), a protein well-established for its importance in creating promoter-enhancer loops (63). In order to establish more directly the role that G4s may have on transcription factor binding and DNA looping, this study utilized three distinct methods of G4 perturbation and subsequently measured associated changes to gene expression within cells. This included: (i) the over-expression of a G4-helicase which conventionally unwinds G4 secondary structures; (ii) the disruption of G4-forming sequences through subtle CRISPR-mediated gene editing; and iii) the use of G4-binding ligands to displace native G4-protein interactions.

In each case, perturbation of G4 formation reduced YY1 binding and looping interactions at G4-sites by an order of magnitude. This was additionally accompanied by significantly altered expression of genes not only directly associated with G4 regions through their promoters, but through distal enhancer sites that interact with said genes via loops as visualized with Hi-C experiments (63). This work is an encouraging example of how it is possible to design experiments to test, in a cellular context, the link between G4s and gene expression, the results of which highlight the currently under-studied proposition that G4s may act as long-distance regulators of transcription.

### G4s may trigger liquid–liquid phase separation events

**Figure 5.**
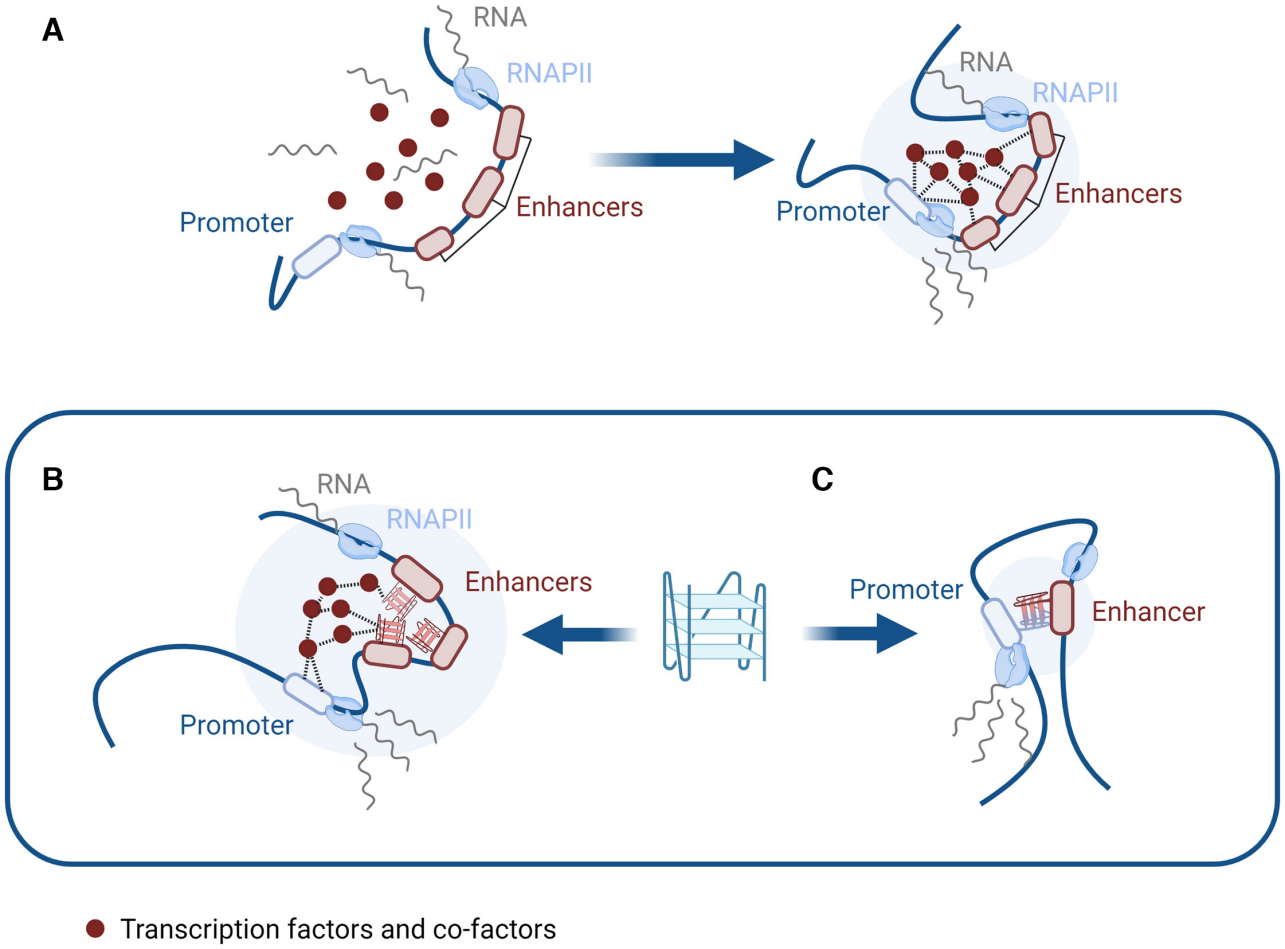
G4s and phase separation. (**A**) Super-enhancers interact with promoter regions via transcription factors, co-factors and chromatin remodelers triggering LLPS that enhances transcription. (**B**) G4s at super-enhancer sites may promote aggregation via intermolecular interactions such as π-stacking between quadruplexes. (**C**) ‘half-G4’ sequences can assemble intermolecularly to mediate enhancer-promoter interactions and phase separation.

In recent years it has been discovered that the expression of genes can be mediated by phase separation events that allow for the concentration of transcriptional machinery within membrane-less organelles inside the nucleus (66,67). Liquid-liquid phase separation (LLPS) relies on a combination of weakly interacting forces between nucleic acids and the low-complexity domains of DNA/RNA-binding proteins (68). This occurs, for instance, in the nucleolus, which is a dense region within the nucleus that contain clusters of ribosomal DNA (69). However, LLPS can also occur outside of the nucleolus in ‘super-enhancer’ regions of the genome characterized by closely localized transcriptional enhancer sequences that bind transcription factors, chromatin remodelling proteins and RNA polymerase II and thus activate transcription (Figure 5A) (70).

This was first demonstrated with the transcriptional co-activator proteins BRD4 (bromodomain-containing protein 4) and MED1 (Mediator 1) which are able to link *via* intermolecular interactions at their disordered domains to create liquid-condensates specifically at super-enhancers (66). The liquid-like state of the BRD4 and MED1 aggregates presents several parallels to membrane-less organelles such as nucleoli and P-granules that are also seen in the nucleus. Additionally, disruption of liquid condensates via treatment with hexanediol, significantly reduced the recruitment of BRD4 and MED1 to super-enhancers (66). As such co-factors are essential for super-enhancer activity, it is thought that the formation of these liquid-condensates may be a unique mechanism to recruit proteins to enhancer regions and achieve transcriptional enhancement.

Although many of the studies considering LLPS in a biological context have historically focused on the protein component of the aggregate, recent work has revealed that certain nucleic acid structures can also act as nucleation sites for phase-separation, even in the absence of proteins. For instance, protein-free aggregation has been observed *in vitro* for RNA repeat expansions, nucleobase homopolymers and some mRNAs by means of RNA–RNA interactions including base pairing, base stacking and other long-range promiscuous interactions (71). Similarly, G4s have the ability to form networks by connecting multiple nucleic acid strands in an intermolecular configuration or by π-stacking between G-tetrads of different G4s, suggesting that they have the potential to cause phase separation exploiting a similar mechanism (Figure 5B). This was exemplified in work directly associating G4s with LLPS in live cells within short root RNA (72) and the C9Orf72 expansion repeat (73). In these studies, it was shown that G4-triggered phase separation is dependent on exposure of the system to G4-favouring conditions, such as increasing potassium concentration, local concentration of nucleic acids and relative guanine content. Furthermore, guanine-rich sequences have been proposed to stimulate the formation of stress granules in the cytoplasm that regulate gene translation, offering yet another piece of evidence to correlate G4 formation to LLPS (74).

The hypothesis that G4s stimulate LLPS by forming intermolecular networks has also been supported by work considering the potential formation of intermolecular G4s within regulatory regions of the genome. For instance, one computational study showed that ‘half-G4’ sequences (containing two instead of four guanine runs) are enriched in promoter and enhancer regions of DNA. It is speculated that when the two guanine runs are brought into close proximity, they are able to hydrogen bond together and thus assemble into a ‘full-G4’, that may mediate promoter-enhancer interactions (75). Similarly, it has been found that regions with a high density of guanine runs are capable of forming quadruplexes over unconventionally long distances and are significantly enriched within super-enhancer regions (76).

Thus, the formation of intermolecular or long-distance G4 structures in such regions may be a mechanism of DNA networking that stimulates LLPS and promotes enhancer activity (Figure 5C). This is particularly relevant in light of our recent findings on the nucleolar protein CSB, which we found to exhibit picomolar affinity for intermolecular G4s whilst displaying negligible binding to intramolecular G4s (77). The characterization of CSB as the first selective intermolecular G4 interactor combined with the known nucleolar localization of this protein suggests that intermolecular G4s may form within the nucleoli and contribute to its phase-separated state, however, this is yet to be explicitly demonstrated.

As such long-range and intermolecular interactions are favoured in crowded environments (78), it is possible that G4s are even more likely to form in the crowded bubbles of liquid condensates (79). Thus, this work brings forward a series of evidence suggesting that G4s might act as nucleation points for LLPS and in turn regulatory elements that may influence transcriptional control. As LLPS offers an interesting mechanism by which the cell increases the local concentration of regulatory proteins and is subsequently able to increase the transcription rate of a given gene, exploring such mechanisms in relation to G4s may yield insightful results. However, investigations into this area are still in their infancy and demonstrate the need for additional work on the diverse roles that physical and chemical changes associated with G4 formation might play in the broader context of gene regulation.

### G4s stimulate expression by promoting DNA oxidation and repair

**Figure 6.**
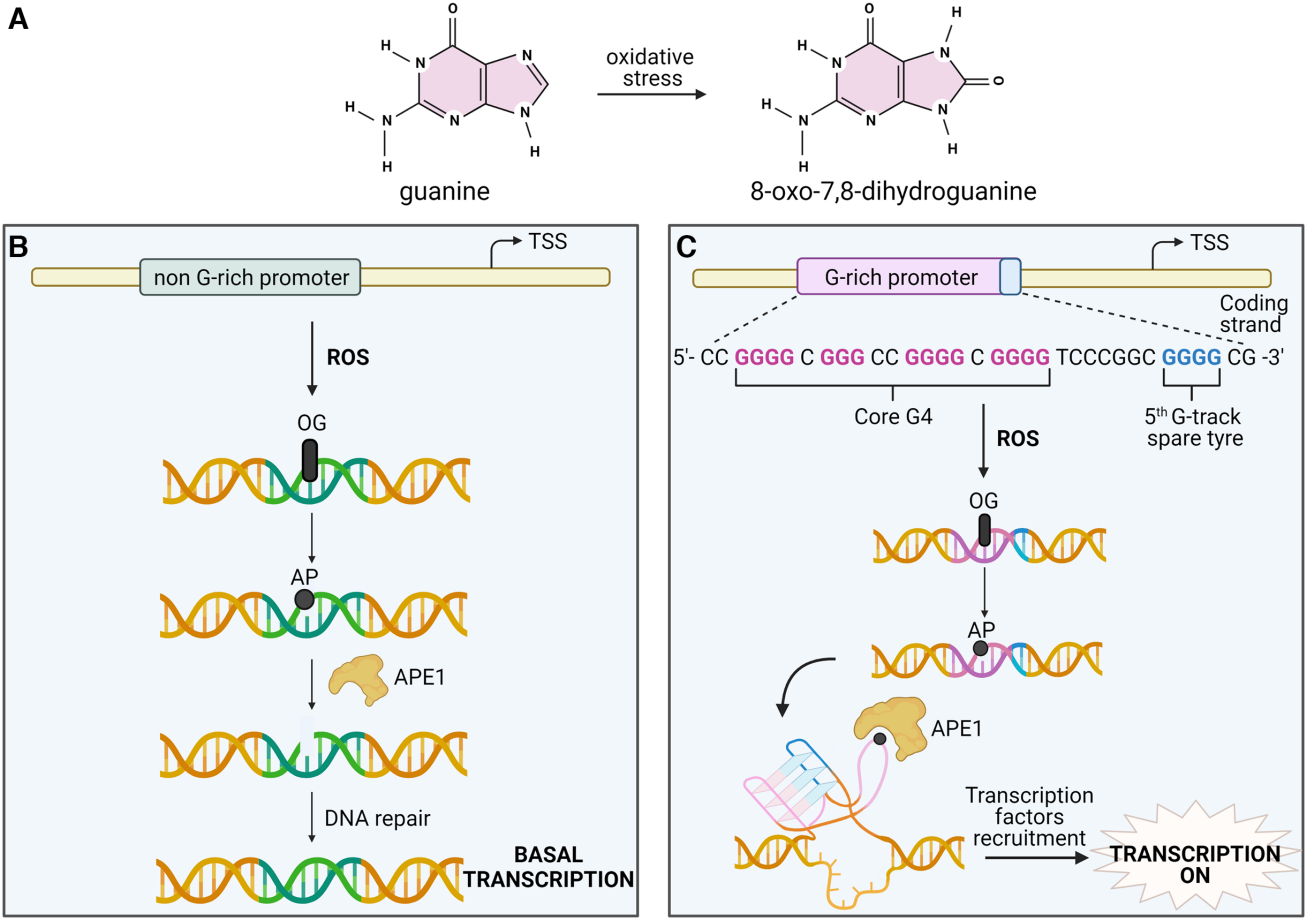
G4s and DNA damage. (**A**) Guanine is frequently oxidized by oxidative stress generating 8-oxo-7,8-dihydroguanine (OG). (**B**) OG formation upon ROS damage in a gene promoter without a G4 causes the formation of an apurinic (AP) site that is recognized and cleaved by APE1. This cleaved site is subsequently repaired without increasing the gene transcription level. (**C**) OG formation in a gene promoter containing a core G4 sequence (in pink) and a fifth G-track (spare tyre in blue) causes the formation of an alternative G4-structure and the extrusion of the AP site into a loop. The structure is recognized and bound by APE1 which reduces the cleaving ability of APE1 and stimulates the recruitment of transcription factors with consequent transcriptional activation.

An additional area in which epigenetic control appears at the G4 level can surprisingly be seen when considering how cells respond to environmental stresses. An example of this stress is demonstrated by guanine oxidation induced by reactive oxygen species (ROS) that arise from multiple exogenous and endogenous factors such as exposure to radiation and metabolism (80,81). Among the four DNA bases, guanine has the lowest redox potential, therefore, guanines are one of the most frequent sites to be oxidized by ROS generating 8-oxo-7,8-dihydroguanine (OG) DNA (Figure 6A) (82,83). Consequently G4s, being composed of several runs of guanines, are easy targets for guanine oxidation (84). As oxidizing species can potentially affect many cellular processes, resulting in ageing or cancer (85,86), exposure to ROS is a constant challenge for cells which have developed efficient repair mechanisms to cope with such mutagenic stress (81). However, the stimulation of these DNA damage repair pathways is not always a negative event and may in fact have important implications for transcriptional control particularly in relation to G4s.

This has been explored in recent studies which demonstrated that OG formation in gene promoters containing G4-forming sequences can stimulate DNA repair mechanisms and promote gene activation (87–89). In one study, a luciferase reporter assay was employed to investigate the effects on gene expression when inserting OG bases into the *VEG*F and *NTHL1* promoters (87). Surprisingly, a 300% increase in luciferase expression was measured with the OG plasmid compared to the plasmid without the oxidized guanine and this activation was related to the formation of G4s in these promoters. Under normal circumstances, the presence of an OG would trigger a DNA damage repair pathway known as base excision repair (BER) in which the oxidised base is removed leaving an apurinic (AP) site in the DNA sequence (90). Subsequently, another BER-related protein known as APE1 would cleave the apurinic region so it can be repaired by downstream polymerase action (Figure 6B). However, in this study it was shown that when this oxidation occurs in a G4-forming region that contains a fifth extra run (‘spare tyre’) of guanines, an alternative G4 structure can form which causes the AP site to be extruded into a loop.

This looped G4 structure is then recognized and bound by APE1, which reduces the cleaving ability of APE1 and instead unmasks a separate function of the protein as a recruiter of transcription factors (Figure 6C) (87,91). Indeed, previous data reported APE1 as a multifunctional enzyme which can form part of a large transcriptional complex which includes HIF-1, STAT3 and CBP/p300 leading to gene activation (92,93). They hypothesize that the accumulation of transcription factors through the ref-1 domain of APE1 explains the transcriptional enhancement that is seen only when this alternative G4 forms in vicinity to OG sites and hinders the usual nuclease function of APE1 (87). In support of this, they did not observe increased luciferase expression when the OG damage was inserted in *VEGF* promoter with only four G-tracks, suggesting that the new G4 conformation cannot be formed once the AP is extruded into a loop. Similar results were also observed in subsequent studies on other gene promoters such as *PCNA*, as well as in studies considering the location and strand dependency of this phenomenon, providing additional examples of the role of G4s as regulators of gene expression upon G oxidation (88,89,94).

Whilst mechanistically informative, a limitation of these studies is the reliance on plasmid systems with synthetically inserted G4-rich promoters which may not be representative of the role of G4s in a cellular and chromatin context. Despite this, similar results were observed when considering plasmids transfected into human glioblastoma cells where guanines were oxidized using the cytokine TNFa which naturally stimulates oxidative stress within cells, suggesting that these findings are consistent even when tested under more physiologically relevant conditions (95). Furthermore, a separate analysis considered endogenously formed cellular G4s using G4 ChIP-Seq and showed a significant enrichment of APE1 at G4 sites within human cells (96). In this study they additionally verified that the binding of APE1 to G4s not only stabilizes the G4 structure but increases the residence time of the protein at sites of DNA damage (96), further consolidating a mechanism linking G4 oxidation with transcriptional enhancement within cells.

In addition to this, APE1 is not the only protein that appears to be recruited to OG DNA by G4s: a recent report showed that oxidized quadruplexes can be bound by and enhance Poly ADP-ribose polymerase-1 (PARP-1) activity (97). Similarly to APE1, PARP-1 is an important effector of the BER pathway which recognizes various DNA lesions (98) and seems to respond to the formation of the looped G4 structure that arises during DNA damage. Additionally, ChIP-qPCR studies within pancreatic cancer cells suggested that OG formation promotes the recruitment of transcription factor proteins MAZ and hnRNP A1 to the G4-rich *KRAS* promoter and may promote the transcription process (99).

Altogether this work demonstrates that an apparently mutagenic event such as the oxidation of guanine bases by ROS, in a G4 context, could actually be considered as a way to epigenetically control gene expression. Such DNA damage coincides with relaxed helical tension and is particularly likely to occur in guanine-rich regions (87), thus promoting the formation of G4s which in turn appear to tune the activity and recruitment of transcriptional regulators. In this way, DNA damage in the form of guanine oxidation at G4-sites might represent a novel mechanism by which G4s naturally contribute towards transcriptional control within cells.

## CONCLUSIONS

In this review, we have explored a number of pathways by which quadruplex DNA may regulate gene transcription with an emphasis on how G4 formation may actually associate with transcriptional activation. This is in direct contrast to the initial and perhaps outdated perspective where G4s are labelled as direct inhibitors of polymerase activity and transcription. Despite this, the goal of this review is not to argue that G4s act solely as transcriptional activators, as there may be individual cases where G4s are repressive in nature (100), but to critically highlight the substantial evidence showing that G4s are globally correlated with transcriptional enhancement rather than repression. This forces us to assess the way in which we have been investigating G4s in terms of gene regulation.

Early studies considered how individual G4s behave within plasmid systems or relied on ligands that artificially stabilize dynamic G4 structures and may additionally displace natural G4-binding partners. This work contributed to a narrative that depicts G4s as transcriptional roadblocks and might not reflect at all the endogenous role of these structures. Such a narrative is still often re-iterated in the current literature and reflects a simplistic view of G4 formation and transcriptional regulation that we strongly feel needs to be updated in light of recent studies. Going forward, it is important to consider the G4 not as an isolated entity within a specific genomic location, but as a structure that exists as part of an interconnected network of other biomolecules in living cells.

Recent research conducted in this spirit has revealed multiple exciting mechanisms that may explain how G4s act as transcriptional regulators. This includes the role of G4s in mediating the placement of histone marks and in interacting with chromatin remodelling proteins, thus shaping the higher order structure of chromatin. Additionally, the notion that G4s can influence the formation and stability of transcriptional loops including R-loops and long-range enhancer-promoter loops has been demonstrated. Even relatively new mechanisms considering intermolecular G4s as triggers of liquid-liquid phase separation in combination with selective interactors (such as CSB) and DNA-damage induced transcriptional activation are being explored. Altogether these novel mechanisms strongly challenge the simple ‘on/off’ switch role that it is still often associated to G4 formation.

By considering G4s as more than simple knot-like impediments for RNA polymerase to overcome, it will be possible to develop these new avenues of research which may be particularly important for investigations of transcriptional dysfunction in pathologies such as cancer and ageing. It follows that further research in this area may unravel a distinct layer of epigenetic regulation in which G4s are implicated as multi-faceted regulatory elements in a complex cellular environment.
